# *RTN2* deficiency results in an autosomal recessive distal motor neuropathy with lower limb spasticity

**DOI:** 10.1093/brain/awae091

**Published:** 2024-03-25

**Authors:** Reza Maroofian, Payam Sarraf, Thomas J O’Brien, Mona Kamel, Arman Cakar, Nour Elkhateeb, Tracy Lau, Siddaramappa Jagdish Patil, Christopher J Record, Alejandro Horga, Miriam Essid, Laila Selim, Hanene Benrhouma, Thouraya Ben Younes, Giovanni Zifarelli, Alistair T Pagnamenta, Peter Bauer, Mukhran Khundadze, Andrea Mirecki, Sara Mahmoud Kamel, Mohamed A Elmonem, Ehsan Ghayoor Karimiani, Yalda Jamshidi, Amaka C Offiah, Alexander M Rossor, Ilhem Ben Youssef-Turki, Christian A Hübner, Pinki Munot, Mary M Reilly, André E X Brown, Sara Nagy, Henry Houlden

**Affiliations:** Centre for Neuromuscular Diseases, UCL Queen Square Institute of Neurology, London WC1N 3BG, UK; Department of Neuromuscular Diseases, Iranian Centre of Neurological Research, Neuroscience Institute, Tehran University of Medical Sciences, Tehran 1416753955, Iran; Department of Neurology, Imam Khomeini Hospital Complex, Tehran University of Medical Sciences, Tehran 1416753955, Iran; Institute of Clinical Sciences, Imperial College London, London SW7 2AZ, UK; MRC Laboratory of Medical Sciences, London W12 0HS, UK; Department of Pediatrics, Neurology and Metabolic Division, Kasr Alainy Faculty of Medicine, Cairo University, Cairo 4240310, Egypt; Centre for Neuromuscular Diseases, UCL Queen Square Institute of Neurology, London WC1N 3BG, UK; Neuromuscular Unit, Istanbul Faculty of Medicine, Istanbul University, Istanbul 34093, Turkey; Department of Clinical Genetics, Cambridge University Hospitals NHS Foundation Trust, Cambridge CB2 0QQ, UK; Centre for Neuromuscular Diseases, UCL Queen Square Institute of Neurology, London WC1N 3BG, UK; Division of Medical Genetics, Mazumdar Shaw Medical Center, Narayana Hrudayalaya Hospital, Bangalore 560099, India; Centre for Neuromuscular Diseases, UCL Queen Square Institute of Neurology, London WC1N 3BG, UK; Centre for Neuromuscular Diseases, UCL Queen Square Institute of Neurology, London WC1N 3BG, UK; LR18SP04, Department of Child and Adolescent Neurology, National Institute Mongi Ben Hmida of Neurology, University of Tunis El Manar, Tunis, 1007, Tunisia; Department of Pediatrics, Neurology and Metabolic Division, Kasr Alainy Faculty of Medicine, Cairo University, Cairo 4240310, Egypt; LR18SP04, Department of Child and Adolescent Neurology, National Institute Mongi Ben Hmida of Neurology, University of Tunis El Manar, Tunis, 1007, Tunisia; LR18SP04, Department of Child and Adolescent Neurology, National Institute Mongi Ben Hmida of Neurology, University of Tunis El Manar, Tunis, 1007, Tunisia; CENTOGENE GmbH, Rostock 18055, Germany; NIHR Oxford Biomedical Research Centre, Centre for Human Genetics, University of Oxford, Oxford OX3 9DU, UK; CENTOGENE GmbH, Rostock 18055, Germany; Institute of Human Genetics, Jena University Hospital, Friedrich Schiller University, Jena 07747, Germany; Institute of Human Genetics, Jena University Hospital, Friedrich Schiller University, Jena 07747, Germany; Department of Radiology, Cairo University, Cairo 12613, Egypt; Department of Clinical and Chemical Pathology, Kasr Alainy Faculty of Medicine, Cairo University, Cairo 12613, Egypt; Molecular and Clinical Sciences Institute, St. George’s, University of London, London SW17 0RE, UK; Innovative Medical Research Center, Mashhad Branch, Islamic Azad University, Mashhad 9187147578, Iran; Molecular and Clinical Sciences Institute, St. George’s, University of London, London SW17 0RE, UK; Division of Clinical Medicine, School of Medicine & Population Health, University of Sheffield, Sheffield S10 2RX, UK; Centre for Neuromuscular Diseases, UCL Queen Square Institute of Neurology, London WC1N 3BG, UK; LR18SP04, Department of Child and Adolescent Neurology, National Institute Mongi Ben Hmida of Neurology, University of Tunis El Manar, Tunis, 1007, Tunisia; Institute of Human Genetics, Jena University Hospital, Friedrich Schiller University, Jena 07747, Germany; Center for Rare Diseases, Jena University Hospital, Friedrich Schiller Universität, Jena 07747, Germany; Dubowitz Neuromuscular Centre, Great Ormond Street Hospital NHS Trust, London WC1N 3JH, UK; Centre for Neuromuscular Diseases, UCL Queen Square Institute of Neurology, London WC1N 3BG, UK; Institute of Clinical Sciences, Imperial College London, London SW7 2AZ, UK; MRC Laboratory of Medical Sciences, London W12 0HS, UK; Centre for Neuromuscular Diseases, UCL Queen Square Institute of Neurology, London WC1N 3BG, UK; Department of Neurology, University Hospital Basel, University of Basel, Basel 4031, Switzerland; Centre for Neuromuscular Diseases, UCL Queen Square Institute of Neurology, London WC1N 3BG, UK

**Keywords:** polyneuropathy, hereditary spastic paraplegia, dHMN, neurodegeneration

## Abstract

Heterozygous *RTN2* variants have been previously identified in a limited cohort of families affected by autosomal dominant spastic paraplegia (SPG12-OMIM:604805) with a variable age of onset. Nevertheless, the definitive validity of SPG12 remains to be confidently confirmed due to the scarcity of supporting evidence.

In this study, we identified and validated seven novel or ultra-rare homozygous loss-of-function *RTN2* variants in 14 individuals from seven consanguineous families with distal hereditary motor neuropathy (dHMN) using exome, genome and Sanger sequencing coupled with deep-phenotyping.

All affected individuals (seven males and seven females, aged 9–50 years) exhibited weakness in the distal upper and lower limbs, lower limb spasticity and hyperreflexia, with onset in the first decade of life. Nerve conduction studies revealed axonal motor neuropathy with neurogenic changes in the electromyography. Despite a slowly progressive disease course, all patients remained ambulatory over a mean disease duration of 19.71 ± 13.70 years. Characterization of *Caenorhabditis elegans RTN2* homologous loss-of-function variants demonstrated morphological and behavioural differences compared with the parental strain. Treatment of the mutant with an endoplasmic/sarcoplasmic reticulum Ca^2+^ reuptake inhibitor (2,5-di-tert-butylhydroquinone) rescued key phenotypic differences, suggesting a potential therapeutic benefit for RTN2-disorder. Despite RTN2 being an endoplasmic reticulum (ER)-resident membrane shaping protein, our analysis of patient fibroblast cells did not find significant alterations in ER structure or the response to ER stress.

Our findings delineate a distinct form of autosomal recessive dHMN with pyramidal features associated with RTN2 deficiency. This phenotype shares similarities with SIGMAR1-related dHMN and Silver-like syndromes, providing valuable insights into the clinical spectrum and potential therapeutic strategies for RTN2-related dHMN.

## Teaser

Maroofian *et al.* delineate a new form of autosomal recessive distal hereditary motor neuropathy (dHMN) with pyramidal features associated with RTN2 deficiency, and conclude that the disorder shares similarities with SIGMAR1-related dHMN and Silver-like syndromes.

## Introduction

*RTN2* (HGNC:10468) encodes RTN2, an endoplasmic reticulum (ER)-shaping protein localized to the ER. In maize, it binds the autophagy protein ATG8a upon ER stress.^1^ However, its role in ER structure is still unclear. Heterozygous *RTN2* variants have previously been associated with progressive hereditary spastic paraplegia (HSP) (SPG12-MIM#604805).^2,3^ However, this association is yet to be confidently established.

To date, the empirical evidence includes information concerning two extended European families, encompassing a minimum of 23 individuals harbouring a segregating ultra-rare founder frameshift variant. Furthermore, there are reports of seven individuals from seven small families with four missense variants, one nonsense variant, one complete gene deletion and two frameshift variants. The reported phenotype is predominantly pure HSP with a varying age of onset ranging from 5 to 50 years old.^2,3^

While the co-segregation of the founder *RTN2* frameshift variant in two independent extended families strongly supports its association with the HSP phenotype on chromosome 19q13, it is important to clarify that this evidence supports the linkage rather than directly proving the pathogenicity of the variant itself. Furthermore, all the reported variants are found in very low frequency in large human genetic variant databases such as gnomAD, UK-Biobank and the UK's 100 000 Genomes Project. This may be attributed to incomplete or low penetrance of the variants or their lack of pathogenicity.

Here, we identified 14 individuals from seven independent consanguineous families with homozygous loss-of-function (LoF) *RTN2* variants presenting with slowly progressive distal motor neuropathy with spasticity and hyperreflexia affecting the upper and lower limbs. Analyses of fibroblasts from a patient homozygous for the *RTN2* p.Gly27Ter did not reveal obvious changes in ER structure or the response to ER stress. By generating a *Caenorhabditis elegans* disease model containing LoF of the worm *RTN2* orthologue, *ret-1*, we identified key behavioural and phenotypic differences in the mutant, which are amenable to drug screening efforts, and confirmed that the inhibition of sarcoplasmic reticulum (SR)/ER Ca^2+^ reuptake may have some therapeutic benefit in the treatment of diseases involving *RTN2* variants.

## Materials and methods

### Patient ascertainment, clinical assessments and genetic investigation

**Figure 1:**
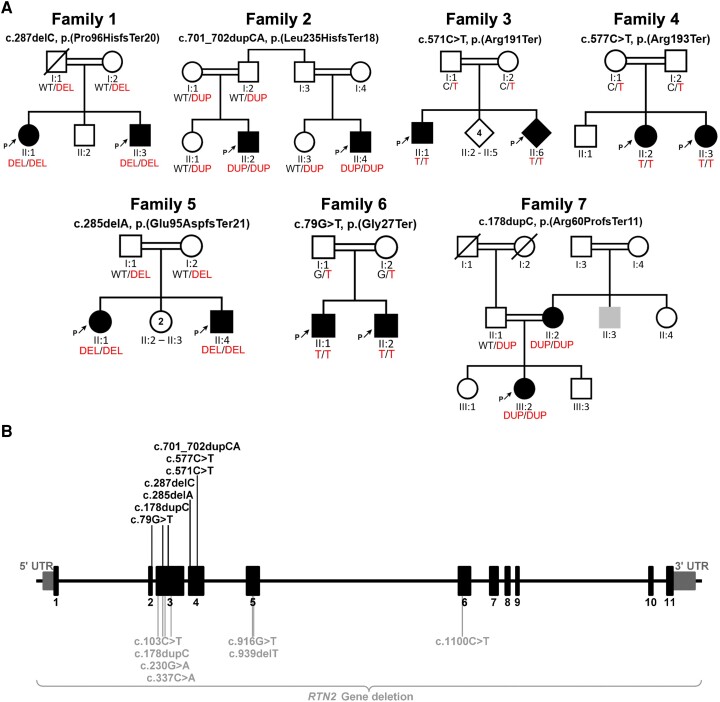
**The illustration depicts family pedigrees and the *RTN2* gene, highlighting the biallelic and monoallelic variants identified in this study and previous studies respectively**. (**A**) Pedigrees of 14 individuals from seven consanguineous families with segregating homozygous *RTN2* variants are shown. (**B**) The localization of the presented variants across the gene is displayed. With homozygous loss-of-function variant (black) identified in this study shown above the gene schematic, and previously reported heterozygous variants (grey) shown below the gene.

Using exome, genome, and Sanger sequencing, along with extensive data sharing with global clinical and research genetics laboratories, we identified 14 individuals (seven males and seven females) from seven consanguineous families harbouring homozygous LoF *RTN2* variants (Fig. 1A). The age range of the affected individuals was 9–35 years for males and 11–50 years for females. Comprehensive data, including detailed clinical descriptions, electrodiagnostic results, brain and muscle imaging, as well as photographic and video documentation of the affected individuals, were collected. All participating families provided written informed consent.

### Cell culture and protein analysis including immunocytochemistry and immunoblotting

Human fibroblasts were cultured in Dulbecco’s Modified Eagle medium (DMEM, Gibco, 31966–021) supplemented with 10% fetal bovine serum (Biowest, S1810-500) and 1% penicillin streptomycin (Gibco, 15070–063) and maintained in a humidified atmosphere under 5% CO_2_ at 37°C.

For immunostaining, cells were seeded on 14 mm coverslips and fixed in 4% paraformaldehyde (PFA) for 15 min. Cells were washed three times, permeabilized with 0.25% Triton X-100 for 10 min and blocked using 5% normal goat serum (BIOZOL, S-1000) for 1 h at room temperature. Coverslips were incubated overnight with primary antibodies: sheep anti-CLIMP63 (1:1000, R&D Systems, AF-7355) and rabbit anti-NOGO (1:1000, Abcam, ab74085) diluted in blocking solution at 4°C. The next day the coverslips were washed three times and incubated with secondary antibodies conjugated with a fluorophore for 1 h at room temperature. After washing three times with 1× phosphate buffered saline (PBS), cells were stained with Hoechst (1:10,000, Invitrogen, H3569) and mounted with fluoromount mounting medium (BIOZOL, SBA-0100-01). Images were acquired with a confocal scanning fluorescence microscope (Zeiss LSM 880) with Airyscan using the Plan-Apochromat 63×/1.4 oil differential interference contrast (DIC) M27 objective and further analysed in ImageJ.

For ER stress induction and the cell viability count, cells were seeded in six-well plates and cultured to 70–80% confluency. Cells were washed with PBS and incubated with new medium supplemented with 5 µg/ml tunicamycin (Santa Cruz) without or in combination with 1 µM MG132 (Calbiochem, 474787-10MG) to inhibit the proteasome. After 48 h, the culture medium was removed, and cells were washed with PBS and trypsinized. All cells were pooled, centrifuged at 800 rpm for 5 min (Heraeus Sepatech Megafuge 2.0R) and resuspended in fresh medium. Cell viability was measured by trypan blue exclusion with an automatic counting device (Bio-Rad TC20 Automatic Cell Counter). For western blot analysis, cells from 10 cm plates were washed twice on ice in ice-cold PBS and lysed directly by adding radioimmunoprecipitation assay (RIPA) lysis buffer. Samples were prepared and subjected to sodium dodecyl-sulfate polyacrylamide gel electrophoresis (SDS-PAGE) and western blotting as described previously.^4^

### *C. elegans* mutant generation and drug response analysis

A homozygous *C. elegans* mutant strain, *ret-* (*syb4955*), containing LoF deletion (17.8 kbp) of the entire coding region of the worm *RTN2* orthologue, *ret-1*, was generated by SunyBiotech (Fuzhou, Fujian, China) using clustered regularly interspaced short palindromic repeats (CRISPR) in an N2 background. Worms were cultured on nematode growth medium at 20°C with *Escherichia coli* strain OP50 following standard methods.^5^ Phenotypic differences in baseline morphology, posture and locomotion of *ret-1*(*syb4955*) and differences in behaviour as the result of treatment with a panel of bioactive molecules were identified using high-resolution worm tracking coupled with automated behavioural phenotyping. Age synchronized populations of young adult worms were reared and imaged using methods previously described in detail.^6^ For drug experiments, worms were exposed to compounds for 4 h prior to imaging (detailed protocol: dx.doi.org/10.17504/protocols.io.dm6gpj19dgzp/v1). Videos were acquired and behavioural features extracted using a high-resolution multi-camera array tracker and Tierspy, following methods previously described in detail.^7,8^

### Ethics

The study was conducted in accordance with the Declaration of Helsinki and approved by the Institutional Review Board at University College London (#310045/1571740/37/598). All participating families provided written informed consent.

## Results

### Clinical phenotyping and characterization

**Figure 2:**
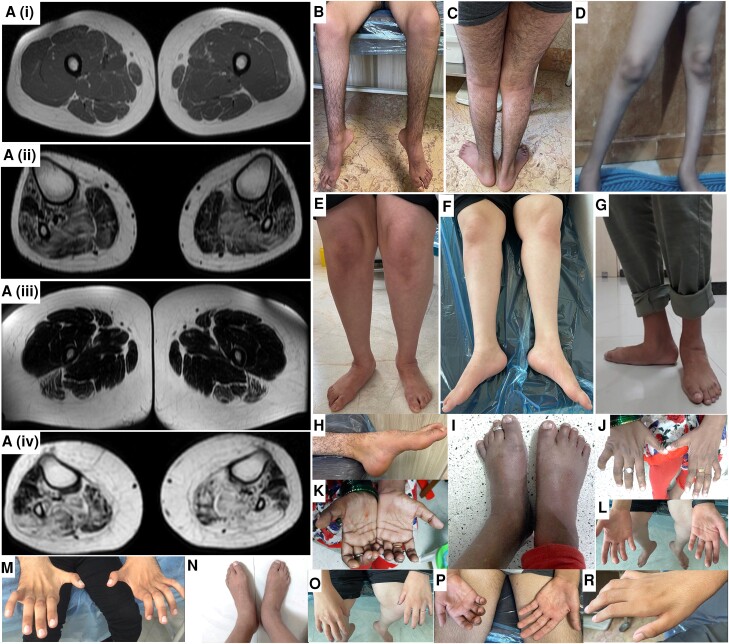
**Images displaying distal limbs and muscle MRI from families with RTN2-related distal hereditary motor neuropathy.** Muscle weakness and wasting of the distal lower > upper limbs in affected individuals from Families 1 (**A–F** and **M–R**), 2 (**G**) and 3 (**H–L**). Axial fat T1-weighted images of the lower limbs in a 17-year-old Iranian boy [**A**(**i** and **ii**)] and his 27-year-old sister from Family 1 [**A**(**iii** and **iv**)]. The thigh muscles of the boy are normal [**A**(**i**)]. There is fatty infiltration of his leg muscles with relative sparing of the medial head of gastrocnemius and extensor digitorum longus [**A**(**ii**)]. Changes are more severe in his older sister with generalized loss of muscle bulk and fatty infiltration of the hamstrings [**A**(**iii**)]. There is more extensive change in her legs, with relative sparing of the medial head of the gastrocnemius, tibialis anterior, extensor digitorum longus and peroneus longus [**A**(**iv**)].

Affected individuals were from consanguineous Iranian (two families), Indian (two families), Egyptian, Tunisian and Pakistani families. One affected sibling of the affected individual from Family 3 did not give consent and was therefore excluded from the detailed analysis (Table 1 and Fig. 2).

**Table 1:** Clinical features of affected individuals with RTN2-related distal hereditary motor neuropathy

	F1		F2		F3		F4		F5		F6		F7	
	Case 1	Case 2	Case 3	Case 4	Case 5	Case 6^a^	Case 7	Case 8	Case 9	Case 10	Case 11	Case 12	Case 13	Case 14
Ethnic origin	Iranian		Iranian		Indian		Tunisian		Indian		Egyptian		Pakistani	
Age of onset	2	2	1	2	5	–	2	6	1–2	2	1–2	1–2	5	∼5
Current age/sex/consanguinity	27/F/Y	17/M/Y	23/M/Y	18/M/Y	28/M/Y	–	11/F/Y	13/F/Y	35/M/Y	45/F/Y	11/M/Y	9/M/Y	16/F/Y	50/F/Y
Presentation	Tiptoe walking		Tiptoe walking		Foot drop, falls, muscle wasting LL	–	Falls, gait disturbance		Gait disturbance, tiptoe walking		Tiptoe walking	Gait disturbance	Falls, foot drop	Tiptoe walking
Ambulatory status	Walks independently		Walks independently		Walks independently	–	Walks independently		Walks independently		Walks independently		Walks independently	
Muscle weakness	Distal LL > UL, foot drop, weak finger extensors		Distal LL > UL, foot drop, weak finger extensors		Distal LL = UL, foot drop	–	Distal LL > UL, foot drop		Distal LL = UL, foot, drop, weak finger extensors	Distal LL = UL Foot drop	Distal LL > distal.UL > Proximal LL Weak finger extensors	Distal LL > distal.UL > Proximal LL Weak finger extensors	Distal LL > distal.UL > Proximal LL Foot drop Weak finger extensors	Distal LL > distal.UL > Proximal LL Foot drop Weak finger extensors
Muscle atrophy	Distal UL and LL		Distal UL and LL		Distal UL and LL	–	Distal UL and LL		Distal UL and LL		Distal UL and LL		Distal LL	
Hyperreflexia	Y		Y		Y	–	Y		Y		Y		Y	
Ankle DTRs	Absent		Absent		Absent	–	Present		Absent		Absent		Absent	
LL spasticity	Y		Y		Y	–	Y		Y		Y		Y	
Babinski sign	Y		Y		Y	–	Y		Y		Y		Y	
Movement abnormalities	Action tremor UL		Action tremor UL		N	–	N		N		N		N	
Gait	Spastic		Spastic		Spastic	–	Spastic		Spastic		Spastic		Spastic	
Sensory deficit	N		N		N	–	N		N		N		N	
Bladder dysfunction	N		N		N	–	N		N		N		Urgency	Urgency
Motor delay	N		N		N	–	Delayed walking (20 months)	N	N	Delayed walking (2 years)	N		Motor delay	
Cognitive impairment	N		N		N	–	N		N		N		N	
Skeletal system	Pes planus		Pes cavus, hammer toes		Pes planus	–	Pes planus, SBD	Pes planus, hyperlordosis, SBD	Pes planus, hammer toes		Pes planus, hammer toes		Pes cavus	
Respiratory system	N		N		N	–	N		Bronchial asthma		N		N	
Bulbar involvement	N		N		N	–	N		N		N	N	N	
Visual system	N		N		Congenital cataract	–	N		N		N		N	
Electrodiagnostic	Axonal motor neuropathy with acute denervation and chronic neurogenic changes		Axonal motor neuropathy with acute denervation and chronic neurogenic changes		Axonal motor-predominant neuropathy with chronic neurogenic changes	–	Normal at 8 years old	Normal at 7 years old	Axonal motor neuropathy		Axonal motor and sensory neuropathy with chronic neurogenic changes	Chronic axonal motor neuropathy	Axonal motor neuropathy with chronic neurogenic changes	Axonal motor neuropathy with chronic neurogenic changes
Muscle MRI	Fatty infiltration, muscle atrophy and oedema in calf muscle compartments		NA		NA		NA (Elevated CK)		NA		Normal		NA	
Brain MRI	Normal		Normal		NA		Normal		NA		Normal		Mildly reduced volume of posterior periventricular white matter and CC splenium	NA
*RTN2* variant	Homozygous c.287del, p.(Pro96HisfsTer20)		Homozygous c.701_702dup, p.(Leu235HisfsTer18)		Homozygous c.571C>T, p.(Arg191Ter)		Homozygous c.577C>T, p.(Arg193Ter)		Homozygous c.285del, p.(Glu95AspfsTer21)		Homozygous c.79G > T, p.(Gly27Ter)		Homozygous c.178dupC, p.(Arg60ProfsTer11)	

CC = corpus collosum; CK = creatine kinase; ENMG = electroneuromyography; F = female; LD = learning difficulties; LL = lower limb; M = male; N = no; NA = not available; SBD = shoulder blades detachment; UL = upper limb; Y = yes.

^a^Not reported details due to refused patient consent.

Consistent features included lower limb spasticity, hyperreflexia, spastic gait and weakness of both the distal upper and lower limbs (lower > upper) with an early age of onset of between 1 and 6 years. Typically, affected individuals presented with early gait disturbances (tiptoe walking, frequent falls). Foot deformities were common and included pes cavus, hammertoes or pes planus. Atrophy of distal upper and lower limb muscles, along with weakness, prominent in finger extensors, was seen in nearly all individuals (Fig. 2B–P). Sensory deficits and cranial nerve involvement were not reported in any cases. Mild motor delay was reported in three individuals and urinary urgency in one.

Nerve conduction studies confirmed axonal motor neuropathy with neurogenic changes on electromyography. In two individuals, sensory nerve conduction in the lower limbs was also reported to be abnormal. Muscle MRI in two affected siblings of Family 1 performed at the ages of 17 and 27 years showed muscle atrophy, inflammation and fatty infiltration, particularly of the calf muscles (Fig. 2A). However, hand and thigh muscle MRI images were normal in two affected siblings from Family 6 at the ages of 9 and 11 years. Brain MRI images from Families 1 and 2 were reported to be normal, while one individual (Individual 13) from Family 7 showed some non-specific neuroimaging findings.

All participants had a slowly progressive disease course and remained ambulatory at the time of the investigation; the oldest participant is currently 50 years of age. None of the affected individuals died prematurely or had any vision, hearing, swallowing or respiratory problems.

While a comprehensive neurological examination was not conducted, it is noteworthy that all parents and carrier siblings exhibited no observable neurological symptoms.

### Genetic findings

Exome and genome sequencing revealed the following homozygous LoF variants in *RTN2* (NM_005619.5): frameshift variants c.287del p.(Pro96HisfsTer20) and c.701_702dup p.(Leu235HisfsTer18) in Families 1 and 2, respectively; a stop-gain variant, c.571C>T p.(Arg191Ter), in Family 3; a stop-gain variant, c.577C>T p.(Arg193Ter), in Family 4; a frameshift variant, c.285del p.(Glu95AspfsTer21), in Family 5; a stop-gain variant, c.79G>T p.(Gly27Ter), in Family 6; and a frameshift variant, c.178dupC p.(Arg60ProfsTer11), in Family 7. Sanger sequencing segregation analysis demonstrated the complete co-segregation of all variants with the affected status within their respective families (Fig. 1A and B). Notably, these variants were located within sizable regions of homozygosity. Furthermore, their presence was notably absent or occurred at extremely low allele frequencies across diverse genetic variant frequency databases, including but not limited to gnomAD (versions V2, V3 and V4), UK Biobank, Queen Square Genomics, Centogene and the 100 000 Genome Project. The aggregate number of alleles assessed across these databases exceeded 2 000 000 (Supplementary Table 1).

### Functional characterization

Because of the prominent role of ER shaping proteins in ER structure, we stained fibroblasts from Patient XYZ, who was homozygous for the *RTN2* p.G27Ter, and control fibroblasts for the ER sheet marker CLIMP63 and the ER tubule marker RTN4 (NOGO)^9^ (Supplementary Fig. 2A–D). Surprisingly, both the CLIMP63- and the NOGO-labelled relative cell areas did not differ between the patient and control cells. In agreement, the abundance of CLIMP63 and NOGO did not differ in the protein lysates of patient and control fibroblasts (Supplementary Fig. 2E and F). Moreover, cell viability did not differ in steady state or upon induction of ER stress with tunicamycin (Supplementary Fig. 2G–I), suggesting that major ER functions remained intact without RTN2.

**Figure 3:**
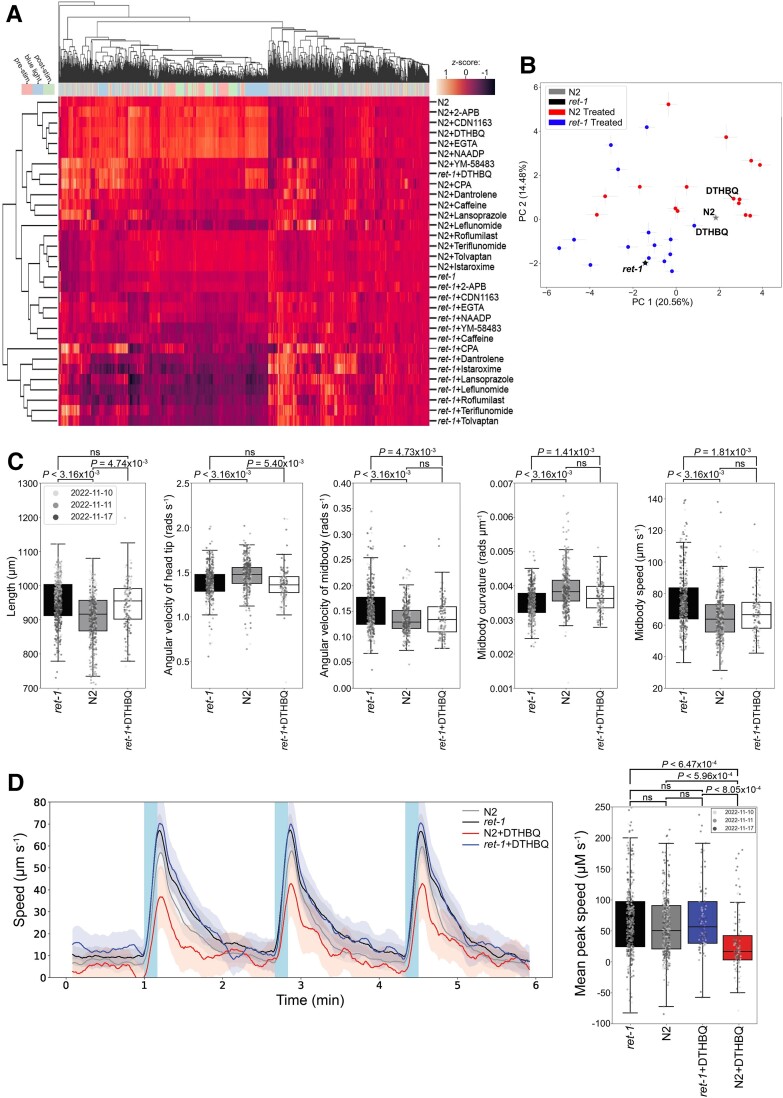
**Loss-of-function ret-1 (RTN2 homologue) *Caenorhabditis elegans* phenotyping and drug screen data.** (**A**) Hierarchical clustering of behavioural fingerprints of untreated wild-type (N2) and *C. elegans* RTN2 orthologue, *ret-1*, loss-of-function (LoF) strains, alongside treatment with the panel of 15 bioactive compounds. The heatmap shows the entire set of 8289 behavioural features extracted by Tierpsy for untreated worms and worms exposed to 100 μM of each compound for 4 h. The top dendrogram shows the relationship of the individual extracted features within the entire feature set, with the tracking period bar denoting when during image acquisition the feature was extracted: pre-stimulation (pink), during stimulation with blue light (blue) and post-stimulation (green). The left dendrogram shows the phenotypic similarity of the worms and the colour map (*top left*) represents the normalized *z*-score of the features. Despite the strains generally clustering separately from each other, treatment of *ret-1* LoF mutant with 2,5-di-tert-butylhydroquinone (DTHBQ) caused this strain to cluster alongside N2, suggesting phenotypic rescue of the mutant. (**B**) Position of N2 and *ret-1* LoF worms in phenospace with respect to the top principal components in the behavioural feature set upon drug treatment. Untreated worms are denoted by stars and treated N2 or treated *ret-1*(*syb4955*). Error bars represent the standard error of the mean. Treatment of the *ret-1* LoF strain with DTHBQ moved the mutant towards the untreated wild-type in phenomic space. (**C**) Key morphological, postural and locomotive features were significantly different between the untreated *ret-1* LoF mutant (black boxes) and untreated N2 (grey boxes). From *left* to *right*: *ret-1* mutants are longer; have decreased angular velocity of the head; increased angular velocity of the midbody; decreased midbody curvature; and increased midbody speed. White boxes show *ret-1*(*syb4955*) exposed to 100 μM DTHBQ for 4 h, rescuing of 3/5 of the key behavioural features. (**D**) Overall speed of *ret-1* LoF mutant and N2 worms after 4 h exposure to DTHBQ. Treatment of N2 with DTHBQ resulted in an attenuation of the photophobic escape response (increased speed) following stimulation of the worms with three 10 s bursts of high-intensity blue light 100 s apart (denoted by blue shaded regions on the line plot), whereas the *ret-1* LoF mutant showed resistance to the effects of DTHBQ. The mean peak speed (box plot, *right*) was calculated as an average of the maximum detected speed across the three independent pulses of blue light. Individual points marked on all box plots are average values from multiple worms (*n* = 3) in a single well. The different point colours indicate data from independent experimental days. *P*-vales were calculated using block permutation *t*-tests (*n* = 10 000 permutations). Permutations were shuffled randomly within, but not between, the independent days of image acquisition in order to control for day-to-day variation in the experiments and corrected for multiple comparisons using the Benjamini-Hochberg procedure to control the false discovery rate at 5%. *P* > 0.05 was considered not statistically significant (ns).

Deletion of the *C. elegans RTN2* orthologue (*ret-1*) caused no developmental delay in worms (data not shown). Comparison of the *ret-1* LoF mutant, *ret-1*(*syb4955*), and the parental strain (N2) revealed significant differences in 3345 morphological, postural and locomotive behavioural features (of 8829 in total) extracted from videos of worms crawling across an agar surface (Fig. 3). Key phenotypic behavioural differences between the strains were as follows: *ret-1*(*syb4955*) worms were longer, had decreased angular velocity of the head, increased angular velocity of the midbody and increased curvature across the body compared with N2. Although there was no significant difference in the overall baseline speed of *ret-1*(*syb4955*) and N2 (data not shown), individual body parts, primarily the head and midbody, exhibited increased speed in *ret-1* LoF worms compared with N2.

We performed a phenotypic comparison of worms treated with a panel of 15 bioactive molecules known to be involved in intracellular calcium signalling pathways (Fig. 3). Hierarchical clustering the behavioural fingerprints of *ret-1*(*syb4955*) and N2 treated with the various compounds (all at a concentration of 100 µM) across the entire feature set revealed five distinct phenotypic clusters (Fig. 3A). There was a defined separation of the parental/mutant strains in phenomic space, although N2 treated with leflunomide or *ret-1*(*syb4955*) treated with cyclopiazonic acid (CPA) clustered as outliers. Treatment with 2-aminoethyl diphenylborinate (2-APB), CDN1163, egtazic acid (EGTA) or nicotinic acid adenine dinucleotide phosphate (NAADP) caused little change in the overall behaviour of wild-type/mutant worms. However, treatment with lansoprazole, leflunomide, roflumilast, teriflunomide, tolvaptan or istaroxime led to large behavioural differences, pushing N2 towards the mutant and *ret-1*(*syb4955*) further away from the parental strain in phenospace.

Importantly, we found that treating the *ret-1* LoF mutant with 2,5-di-tert-butylhydroquinone (DTBHQ), an ER/SR Ca^2+^ reuptake inhibitor, caused the strain to cluster closer to N2 (Fig. 3A), resulting in a reduced number of significant behavioural differences between the mutant and N2 (1072 out of 8289). This finding was further supported by the top principal components extracted from the behavioural feature set (Fig. 3B), where there was clear movement of the *ret-1* LoF mutant towards untreated N2 with respect to both principal components. Furthermore, we note that treatment with DTHBQ rescued 3/5 of the key phenotypic differences observed between N2 and *ret-1*(*syb4955*) (Fig. 3C). We noted that treatment of N2 with DTHBQ caused significant attenuation of the photophobic escape response (defined as an increase in speed following exposure of worms to bursts of high intensity blue light), whereas there was no significant difference in the photophobic escape response of untreated N2/*ret-1*(*syb4955*) or the mutant strain treated with DTHBQ (Fig. 3D). We also noted that the *ret-1* LoF mutant demonstrated resistance to severe attenuation of the photophobic escape response upon treatment with 2-APB [an inositol trisphosphate (IP3) receptor and transient receptor potential (TRP) channel inhibitor; Supplementary Fig. 1]. Overall, these findings demonstrated clear movement of the disease model strain towards the healthy parental strain in overall phenospace upon treatment with DTHBQ.

## Discussion

The distal hereditary motor neuropathies represent a clinically and genetically heterogeneous spectrum of rare neuromuscular disorders characterized by length-dependent predominantly motor neuropathy, manifesting as distal muscle weakness and atrophy.^10-13^ This spectrum is proposed to exhibit a continuum rather than discrete entities, demonstrating both clinical and genetic overlap with axonal Charcot-Marie-Tooth disease (CMT2), juvenile amyotrophic lateral sclerosis, HSP, spinocerebellar ataxia and, in certain instances, distal myopathy. These observations suggested a potential shared molecular pathogenesis among these hereditary neurological disorders.^10-13^

Variable pyramidal tract signs of hyperreflexia and spasticity with distal motor neuropathy can be found in association with pathogenic variants in several genes.^10-12^ Monoallelic pathogenic variants in *BSCL2* (HGNC:15832) encoding an ER membrane protein lead to a wide spectrum of highly variable neurological disorders ranging from pure dHMN type V without spasticity and CMT2 to Silver syndrome (*SPG17*-MIM#270685) and ‘juvenile amyotrophic lateral sclerosis-mimics’.^14^ Monoallelic pathogenic *SETX* (HGNC:445; MIM#608465) variants can also manifest as dHMN accompanied with pyramidal signs.^15^

Additionally, heterozygous pathogenic variants of *KIF5A* (HGNC:6323), *SPAST* (HGNC:11233) and *ATL1* (HGNC:11231) can lead to Silver-like syndrome presentations in SPG10, SPG4 and SPG3A, respectively.^16-18^*REEP1* (HGNC:25786) variants are found in patients who occasionally present with features overlapping with dHMN-V. In these cases, early and selective involvement of the thenar and dorsalis interosseus I muscle can be seen.^19^ Spasticity can also rarely be observed in dominant dHMN caused by pathogenic variants of *HSPB1* (HGNC:5246).^20^

Biallelic pathogenic variants of *SIGMAR1* (HGNC:8157), encoding a transmembrane ER protein involved in ER-mitochondrial signalling, have recently been associated with autosomal recessive dHMN and Silver-like syndrome in several families.^21-23^ The phenotype associated with *SIGMAR1*, and one of the most frequent HSP subtypes, *SPG11* (HGNC:11226), can also mimic juvenile amyotrophic lateral sclerosis and might be misdiagnosed as such.^21,24^ Furthermore, biallelic *COQ7* (HGNC:2244) variants were also recently described to cause dHMN with and without pyramidal signs.^25^

In this report, we have delineated a new subtype of recessive dHMN with pyramidal features associated with RTN2 deficiency. The distinct and unusual clinical feature of the novel RTN2-disorder is finger extensor weakness, mimicking the phenotype of SIGMAR1-disorder. Furthermore, the phenotypic presentation mimics Silver-like diseases. However, in contrast to patients with biallelic variants in *SIGMAR1*, those with biallelic *RTN2* pathogenic variants have a consistent spastic involvement of the legs in early childhood. Furthermore, unlike Silver-like syndromes, all affected individuals (apart from one) showed a predominantly distal lower limb weakness with early foot deformities and gait abnormalities at disease onset instead of early upper limb involvement. Interestingly, none of the heterozygous family members had any symptoms related to spasticity or neuropathy, which might be explained by incomplete or very low penetrance or a controversial role of the variants in the monoallelic state.

The muscle MRI findings in four individuals reported here, suggested an imaging phenotype consistent with length-dependent polyneuropathies in older individuals, specifically greater lower than upper limb involvement and more severe involvement distally. In the thighs, the hamstrings appeared to be involved first, while in the lower legs, the medial head of gastrocnemius and extensor digitorum longus were relatively spared. The imaging changes progressed with age and disease stage, with normal findings in the affected individuals aged 9 and 11 years from Family 6 and relatively mild changes and more severe changes in the individuals aged 17 years and 27 years from Family 1, respectively. However, confirmation of any phenotype/genotype correlation will require the analysis of MRI images from more patients.

The exact pathogenicity of *RTN2-*related disorder is poorly understood. RTN2 is known to be localized to the ER and involved in the dynamic remodelling of the ER.^1,4^ In addition, RTN2 interacts with FAM134B, a protein mediating ER-phagy. FAM134B LoF is associated ER expansion and the accumulation of misfolded proteins, and it sensitizes cells to undergo apoptosis and causes recessive hereditary sensory and autonomic neuropathy type IIB, which can present with motor symptoms such as spasticity and distal weakness.^4,26-28^ Our preliminary analysis, however, did not unveil major alterations to the ER structure or the response to ER stress as previously observed with FAM134B LoF.^4,27^

The ER plays critical roles in several cellular processes, including calcium homeostasis, and several monogenic neurodegenerative disorders, particularly those affecting upper or lower motor neurons with a variable degree of sensory involvement, have been associated with pathogenic variants in genes including *ATL1*, *ATL3* (HGNC:24526), *REEP1*, *REEP2* (HGNC:17975), *SPAST*, *RTN2*, *ARL6IP1* (HGNC:697) and *LNPK* (HGNC:21610), which encode ER-resident membrane-shaping proteins characterized by reticulon homology domains.^4,29^ More recently, deletion of the *Drosophila RTN2* orthologue was shown to alter ER organization and the function of presynaptic terminals significantly.^30^ Despite little change in resting Ca^2+^ storage capacity, this variant led to major reductions in activity-evoked Ca^2+^ fluxes in the cytosol, ER lumen and mitochondria. Interestingly, cytosolic and mitochondrial Ca^2+^ responses in transheterozygous larvae were similar to those in larvae harbouring the wild-type *RTN2* orthologue, further suggesting a pathogenic role for total loss of the protein.

Here, we generated a *C. elegans RTN2* orthologous (*ret-1*) LoF mutant and characterized morphological and behavioural differences, which could be rescued via inhibition of ER/SR Ca^2+^ reuptake. These data support findings from *Drosophila* functional studies that suggest changes in Ca^2+^ signalling pathways contribute towards HSP pathology,^30^ and as such we identified a potential benefit of inhibiting Ca^2+^ reuptake in the ER/SR for the treatment of RTN2-related disorders. Moreover, characterization of the behavioural differences of *C. elegans ret-1* LoF mutants means they are well suited for future high-throughput compound screens to further elucidate the molecular underpinnings of this rare neurological disorder and identify additional candidate targets for treatment. Our findings delineate a new form of autosomal recessive dHMN with pyramidal features associated with RTN2 deficiency.

## Supplementary Material

awae091_Supplementary_Data

## Data Availability

Clinical or functional data may be shared with any qualified investigator upon request. Genome sequencing data for Family 5 is available in the National Genomic Research Library (https://doi.org/10.6084/m9.figshare.4530893.v7).
